# Radio-adaptive response and correlation of non-homologous end joining repair gene polymorphisms [XRRC5 (3R/2R/1R/0R), XRCC6(C/G) and XRCC7 (G/T)] in human peripheral blood mononuclear cells exposed to gamma radiation

**DOI:** 10.1186/s41021-021-00176-4

**Published:** 2021-03-08

**Authors:** Shridevi Shelke, Birajalaxmi Das

**Affiliations:** 1 Low Level Radiation Research Section, Radiation Biology & Health Sciences Division, Bio-Sciences Group, Bhabha Atomic Research Centre, Trombay, Mumbai, 400 085 India; 2 Homi Bhabha National Institute (HBNI), Anushaktinagar, Mumbai, 400094 India

**Keywords:** Radio-adaptive response, DNA double strand breaks, Non-homologous end joining (NHEJ) repair gene polymorphism, Genotypes

## Abstract

**Background:**

Radio-adaptive response (RAR) is transient phenomena, where cells conditioned with a small dose (priming) of ionizing radiation shows significantly reduced DNA damage with a subsequent high challenging dose. The role of DNA double strand break repair gene polymorphism in RAR is not known. In the present study attempt was made to find out the influence of NHEJ repair gene polymorphisms [a VNTR; *XRCC5* (3R/2R/1R/0R); two single nucleotide polymorphisms (SNPs); *XRCC6* (C/G) and *XRCC7* (G/T)] with DNA damage, repair and mRNA expression in human PBMCs in dose and adaptive response studies. Genomic DNA extracted from venous blood samples of 20 random healthy donors (16 adaptive and 4 non-adaptive) and genotyping of NHEJ repair genes was carried out using PCR amplified length polymorphism.

**Results:**

The dose response study revealed significant positive correlation of genotypes at *XRRC5* (3R/2R/1R/0R), *XRCC6*(C/G) and *XRCC7* (G/T) with DNA damage. Donors having genotypes with 2R allele at *XRCC5* showed significant positive correlation with mRNA expression level (0R/2R: *r* = 0.846, *P* = 0.034; 1R/2R: *r* = 0.698, *P* = 0.0001 and 2R/2R: *r* = 0.831, *P* = 0.0001) for dose response. Genotypes C/C and C/G of *XRCC6* showed a significant positive correlation (*P* = 0.0001), whereas, genotype T/T of *XRCC7* showed significant negative correlation (*r* = − 0.376, *P* = 0.041) with mRNA expression.

**Conclusion:**

Interestingly, adaptive donors having C/G genotype of *XRCC6* showed significantly higher (*P* < 0.05) mRNA expression level in primed cells suggesting their role in RAR. In addition, NHEJ repair gene polymorphisms play crucial role with radio-sensitivity and RAR in human PBMCs.

## Introduction

Environmental exposures such as chemical and physical mutagens including ionizing radiation may (IR) pose concern to human health. However, it is important to understand the underlying biological mechanisms regarding the individual susceptibility to radiation exposure. IR induces a variety of DNA damages/lesions such as single strand breaks (SSBs), double strand breaks (DSB) and base damages in human cells in addition to the basal endogenous DNA damages produced by oxidative stress. Radiation induced SSBs and oxidative DNA damages produced in a cell are large in number per 1.0 Gy of low Linear energy transfer (LET) IR, as compared to DNA DSBs, which are very few in number [1]. However, DSBs are most lethal to the cells leading either cell death or may cause adverse consequences leading to genomic instability and carcinogenesis, if not repaired or mis-repaired. Very often, mis-repaired or defective cells accumulate lethal mutations, rearrangement of complex chromosomal aberrations or chromothripsis [2, 3]. These changes activate DNA damage response (DDR), cell cycle check points, and cell survival pathways along with molecular pathways of DNA repair. Radio-adaptive response (RAR) occurs in cells/tissues, when an exposure of low dose (priming dose) of IR helps in reducing the damage caused by a single high dose (challenging dose) of IR [4]. RAR is observed in human cells with a priming dose of IR between (0.001–0.5 Gy) and the challenging dose between (0.1–5.0 Gy) with incubation time between (2-24 h) [3]. There are reports which demonstrate that circulating lymphocytes of individuals show differential response to a high challenging dose. There are two groups of donors i.e., adaptive and non-adaptive, depending upon radio-sensitivity or radio-resistance of individuals. However, it is important to understand the underlying mechanism regarding the genetic basis of this variation in response to IR. Recently, association of base excision repair (BER) gene polymorphisms in RAR has been reported in human peripheral blood mononuclear cells exposed to gamma radiation [5].

Non homologous end joining (NHEJ) pathway of DSB repair is one of the major repair pathways of choice in circulating lymphocytes which is in G_0_/G_1_ phase of the cell cycle [6]. DNA polymorphism such as studies on single nucleotide polymorphisms (SNPs) and variable number of tandem repeats (VNTRs) at NHEJ repair genes (*XRCC5*, *XRCC6* and *XRCC7)* are associated with an increased risk of radiation sensitivity and cancer [7]. Deficiency in DNA repair increases radio-sensitivity in many cancers and human diseases [8]. DNA repair deficient syndromes such as xeroderma pigmentosum (XP), cockayne syndrome (CS), fanconi anemia (FA) and nijmegen breakage syndrome (NBS) are very often show radio-sensitivity. Hence, studying association of genetic variants such as SNPs and VNTRs of DNA repair genes may contribute to the susceptibility to a particular disease or radiation sensitivity [9]. There are studies which suggest that SNPs from DSB repair pathway genes may modulate gamma-radiation induced mutagen sensitivity [10]. Recently, we have also reported that IR influences DNA repair gene polymorphisms and individual radio-sensitivity [5, 11].

The presence of polymorphic alleles in DNA repair genes may alter the repair capacity of a particular individual thus affecting individual susceptibility in developing cancer [12]. It has also been reported that individuals showing severely compromised repair capacity have increased mutation rates, genomic instability, and an increased risk of cancer [13]. Healthy individuals differ in their intrinsic capacity to repair [14], which could be due to alterations in gene expression and association with DNA repair gene polymorphisms.

Low-dose ionizing radiation (LDIR) exposure below 100 mGy (0.1 Gy) may induce RAR and protects the cells from DNA damage, repair and cell killing. However, DNA DSBs are repaired through DSB repair pathways in order to keep the cells maintain genomic integrity [15]. Both homologous recombination repair (HRR) or non-homologous end-joining (NHEJ) repair mechanisms are activated due to LDIR exposure in cell lines in culture, but NHEJ is more active in resting PBMCs. Recent studies from high level natural background radiation areas have shown efficient repair of DSBs and activation of DDR, NHEJ, HR and other signaling pathways in PBMCs at low lose radiation exposed human population [16, 17]. Evidences of the involvement DDR and BER repair pathway in human PBMCs have also been found at DNA damage, repair and mRNA expression level [18–20]. Our earlier studies showed variability in radio-adaptive response in NHEJ repair pathway among the healthy donors [21].

**Fig. 1:**
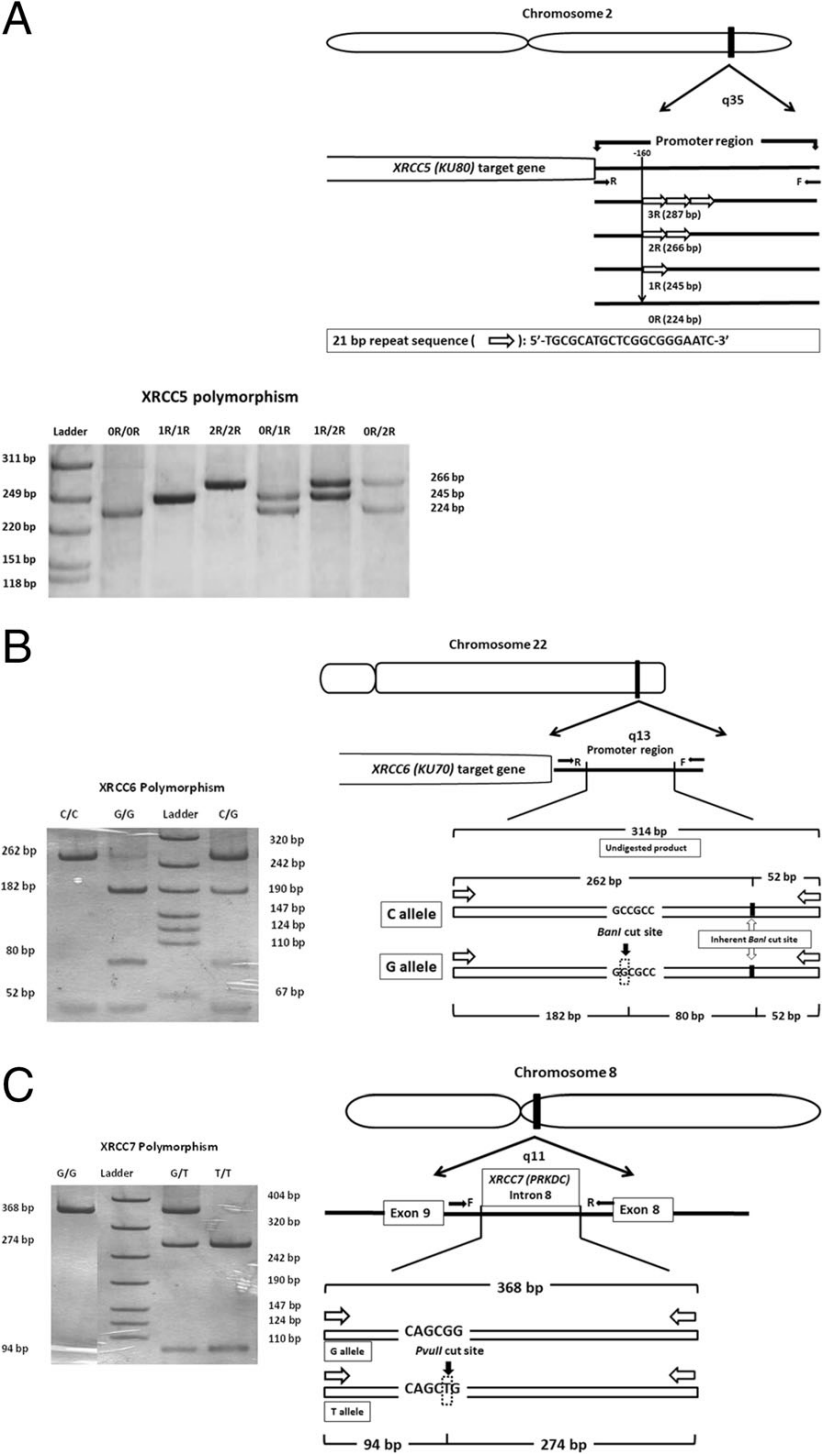
Gene mapping and Silver stained PAGE (6%). **a** *XRCC5* (*KU80*) - 3R/2R/1R/0R VNTR polymorphism. **b** *XRCC6* (*KU70*) – 61C/G. **c** *XRCC7* (*PRKDC*) – G/T6721G/T. Ladder in Fig. **a** & **c**: ɸX174 HinfI digest (Banglore Genei India Pvt. Ltd.); ladder used in Fig. **b**: DNA molecular weight marker VIII (Roche diagnostic Gmbh, Germany)

In the present study, attempt was made to study genetic polymorphism of *XRCC5* (3R/2R/1R/0R), *XRCC6* (C/G) and *XRCC7* (G/T) from NHEJ repair pathway and their possible association with DNA damage and mRNA expression profile among healthy donors. The role of different genotypes in RAR is also explored. Schematic representation of the gene mapping for these polymorphisms are given in Fig. 1. The characteristics of these genes are as follows:

***XRCC5*** **(*KU80*)-3R/2R/1R/0R** is a novel VNTR located in the promoter region of *XRCC5* gene [22, 23]. It displays four different alleles with repetitive sequences of 21 nucleotides repeats (3R, 2R, 1R and 0R). For instance, 3R refers to three 21 nucleotides repeats (3R) (− 221 to − 160 relative to the initiation of Transcription), 2R refers to two 21 nucleotides repeats (2R) (− 201 to − 160), 1R refers to one 21 nucleotides repeat (1R) (− 180 to − 160), and a zero repeat (0R). This polymorphism is found to be associated with acute myeloid leukemia (AML), chronic myeloid leukemia (CML) [23–25]. ***XRCC6*** **(*KU70*)-61C/G** polymorphism is located in the promoter region of chromosome 22. It is also known to be associated with increased risk of breast cancer [26] and AML [23]. Genetic variants of ***XRCC7*** **(*PRKDC*)-6721G/T** is located in the intron 8 of chromosome 8 and has shown to elevate the risk of glioma and renal cell carcinoma [27, 28].

DNA repair polymorphisms are influenced by several susceptibility factors including environmental exposures, which can affect genome integrity and thus can be used as biomarkers of cancer risk in human population [29]. Genetic polymorphisms may affect genotype-phenotype correlation, but not yet established for most of the polymorphisms [30]. Hence, in recent years, efforts are made to develop possible biomarkers of DNA repair gene polymorphisms for efficient detection, treatment, and prevention of human diseases including cancer [31].

The objective of the present study was to find out the correlation of genotypes observed at three NHEJ repair gene polymorphisms [*XRCC5* (3R/2R/1R/0R), *XRCC6* (C/G) and *XRCC7* (G/T)] with respect DNA damage, DNA repair capacity (DRC) and mRNA expression and their influence in RAR.

## Materials and methods

### Ethic statement

Venous blood samples were collected from 20 random, normal and healthy donors (males, Age: 25 to 40 years, non-smokers) in EDTA containing vials with written informed consent, which was approved by Medical ethics committee, Trombay, BARC, Mumbai.

### Collection of blood samples, separation of PBMCs, irradiation and sample preparation

In the present study, approximately 12 ml blood from each donor was collected, PBMCs were separated through gradient centrifugation by overlaying whole blood on Histopaque-1077 (Sigma Aldrich, USA) solution, centrifugation at 400X g for 30 min at room temperature. The layer containing PBMC was removed and washed twice with phosphate-buffered saline and used for DNA damage and mRNA expression studies. Dose response, time point kinetics and RAR studies were conducted using DNA damage, repair and mRNA expression profile.

For dose response study, PBMCs were irradiated at various doses i.e., 0.1, 0.3, 0.6, 1.0 and 2.0 Gy of gamma radiation using ^60^Co γ-radiation source (Bhabhatron II, Panacea Medical Technologies, Bangalore, India) at a dose rate of 1.0 Gy/min. Post irradiation time point kinetics study for quantitation of DSBs and time dependent changes of mRNA expression profile were determined at 2.0 Gy in PBMCs at different time points (30 min, 60 min, 120 min and 240 min) along with un-irradiated control. For all the experiments, un-irradiated PBMCs were simultaneously processed as a control along with the irradiated samples. Radio-adaptive response study was carried out where PBMCs were exposed in vitro with a priming dose of 0.1 Gy followed by 2.0 Gy of gamma radiation after 4 h incubation and analysed for DNA damage, post irradiation time point kinetics and mRNA expression profile. Aliquots of PBMCs (~ 10^6^ cells/ml) were prepared in duplicates for each dose and time point to quantitate DNA damage, repair kinetics and mRNA expression profile.

#### Measurement of DNA DSBs by neutral comet assay

Neutral single cell gel electrophoresis was carried out for quantitation of DNA DSB, post-irradiation changes and RAR study as described elsewhere [21]**.** About 100 cells (50 cells from each slide) were randomly selected and quantified using TriTek Comet Score TM Version 1.5. The percentage of DNA in the tail was calculated as per the calculation as follows:

$$ \%\mathrm{DNA}\ \mathrm{in}\ \mathrm{Tail}=\left[\frac{\mathrm{Total}\ \mathrm{Tail}\ \mathrm{Intensity}}{\mathrm{Total}\ \mathrm{comet}\ \mathrm{Intensity}}\right]\times 100 $$

#### Analysis of mRNA expression by real time q-PCR

Total RNA was extracted and cDNA was prepared from irradiated as well as sham irradiated control PBMCs. Analysis of mRNA expression pattern of the NHEJ repair genes [*XRCC5* (*KU80*), *XRCC6* (*KU70*) and *XRCC7* (*PRKDC*)] was carried out using SYBR green based real time q-PCR for dose response, post irradiation time point kinetics and RAR studies. Details of the primer sequences and PCR cycling conditions were as described elsewhere [21]**.** The results are expressed in normalized ratio as described by Pfaffle et al. (2001) [32] and the calculation is as follows:

$$ \mathrm{Normalized}\ \mathrm{ratio}=\frac{{\left(\mathrm{Concentration}\ \mathrm{of}\ \mathrm{Target}/\mathrm{Concentration}\ \mathrm{of}\ \mathrm{reference}\right)}_{\mathrm{sample}}}{{\left(\mathrm{Concentration}\ \mathrm{of}\ \mathrm{Target}/\mathrm{Concentration}\ \mathrm{of}\ \mathrm{reference}\right)}_{\mathrm{Calibrator}}} $$

#### Genetic polymorphism study

Approximately 2 ml of venous blood samples were collected in EDTA containing vaccutainers from each donor. Genomic DNA was extracted from whole blood using non-enzymatic salt precipitation method [33]. DNA was quantified by taking absorbance at 260 nm and 280 nm using Picodrop Microlitre Spectrophotometer (Pico100, Picodrop Ltd., UK). The ratio of absorbance at 260 and 280 nm was determined to check for the purity of DNA.

#### PCR amplified fragment length polymorphism

Genetic polymorphisms were studied at selected SNPs for *XRCC6* (C/G), *XRCC7* (G/T) and a VNTR at *XRCC5* (3R/2R/1R/0R). The SNPs of *XRCC6* (C/G), and *XRCC7* (G/T) polymorphism were analyzed by PCR amplified restriction fragment length polymorphism (PCR-RFLP). The VNTR at *XRCC5* (3R/2R/1R/0R) polymorphism was studied by PCR amplified fragment length polymorphism (Amp-FLP). All the PCR reactions were performed on Master cycler gradient thermocycler (Eppendorf, Hamburg, Germany) in a final volume of 25 μl containing locus specific primers (5 picomoles of each primer), 50 ng genomic DNA, 1.5 mM MgCl_2_, 200 μmoles each dNTPs and 0.5 unit of Taq DNA Polymerase. Primer sequences and PCR conditions used for polymorphism study are given in Table 1 and Table 2 respectively.

**Table 1:** Locus specific primer sequences of *XRCC5, XRCC6 and XRCC7* genes used for polymorphism study

Name of the primer	Primer sequence	bp
*XRCC5*(*KU80*) PR-1 (forward)	5′-AGGCGGCTCAAACACCACAC-3’	20
*XRCC5*(*KU80*) PR-2 (reverse)	5′-CAAGCGGCAGATAGCGGAAAG-3’	21
*XRCC6* (*KU70*) PR-1(forward)	5′-TCTCCACTCGGCTTTTCTTCCA-3’	22
*XRCC6* (*KU70*) PR-2 (reverse)	5′-TCTCCCTCCGCTTCGCACTC-3’	20
*XRCC7*(*PRKDC*) PR-1 (forward)	5′-CGGCTGCCAACGTTCTTTCC-3’	20
*XRCC7*(*PRKDC*) PR-2 (reverse)	5′-GACATTTTTGTCAGCCAATCTTT-3’	20

**Table 2:** PCR conditions used for polymorphisms studied

		***XRCC5*** (***KU80***)		***XRCC6*** (***KU70***)		***XRCC7*** (***PRKDC***)	
		Temp	Time	Temp	Time	Temp	Time
**Initial denaturation**		95 °C	5 min	95 °C	5 min	95 °C	5 min
**Amplification (30 cycles)**	**Annealing**	60 °C	30s	58 °C	30s	63 °C	30s
	**Extension**	72 °C	30s	72 °C	30s	72 °C	30s
	**Denaturation**	95 °C	30s	95 °C	30s	95 °C	30s
**Final extension**		72 °C	5 min	72 °C	5 min	72 °C	5 min

#### Restriction digestion of PCR amplified products

Approximately 8 μl of amplicons were digested overnight with BanI and PvuII restriction endonucleases (New England Biolabs Inc., UK) at 37 °C for *XRCC6(*C/G) and *XRCC7*(G/T) polymorphisms, respectively. Undigested and restriction enzyme (RE) digested PCR product sizes for *XRCC5* (0R/1R/2R/3R), *XRCC6 (*C/G) and *XRCC7* (G/T) polymorphisms are given in Table 3. The RE digested PCR products were resolved on 10% nondenaturing polyacrylamide gels (PAGE) followed by silver staining (Perkin Elmer method). Silver stained gels with different allele sizes (bps) for each polymorphism (*XRCC5, XRCC6* and *XRCC7)* are given in Fig. 1.

**Table 3:** Restriction enzymes and fragment sizes of polymorphisms studied

Gene Name	SNP/ polymorphism	Enzyme	Genotype	Undigested product (bp)	Digested product (bp)
***XRCC5*** **(*KU80*)**	3R/2R/1R/0R (rs6147172)	–	0R/0R 1R/1R 2R/2R 3R/3R	224 245 266 287	–
***XRCC6*** **(*KU70*)**	61C/ G	BanI	C/C G/G	314	262 and 52 182, 80and 52
***XRCC7*** **(*PRKDC*)**	6721G/T (rs7003908)	PvuII	G/G T/T	368	368 274 and 94

### Statistical analysis

Linear regression analysis was performed to study dose response at DNA damage. For mRNA expression analysis, the normalized ratio was calculated as described by Pfaffle (2001) [32]. Pearson’s correlation coefficient was calculated to determine the correlation between various genotypes with DNA damage and mRNA expression profile. All the statistical analysis was performed using SPSS software (version 17.0). For all the experiments, *P* ≤ 0.05 was set for significance level.

## Results

DNA polymorphism study of *XRCC5* (*KU80*), *XRCC6* (*KU70*) and *XRCC7* (*PRKDC*) genes were carried out among 20 donors and the genotypes were correlated with the DNA damage, repair and transcriptional profile. Linear regression was carried out for dose response study at DNA damage and gene expression level at each of the genotypes to find out the association of polymorphisms at these above genes with DNA damage, mRNA expression and inter-individual variation if any. RAR study was also carried out in primed and non-primed cells of 16 adaptive and 4 non-adaptive donors, where genotypes observed at NHEJ polymorphisms were correlated with biological end points such as DNA damage, repair and mRNA expression.

### Genotype frequencies of NHEJ polymorphism and correlation with DNA damage/repair and mRNA expression

In the present study, the distribution of genotype frequencies at each of the polymorphisms was determined among 20 donors studied and correlated with DNA damage, repair and mRNA expression level.

### *XRCC5* (*KU80*) polymorphism

**Fig. 2:**
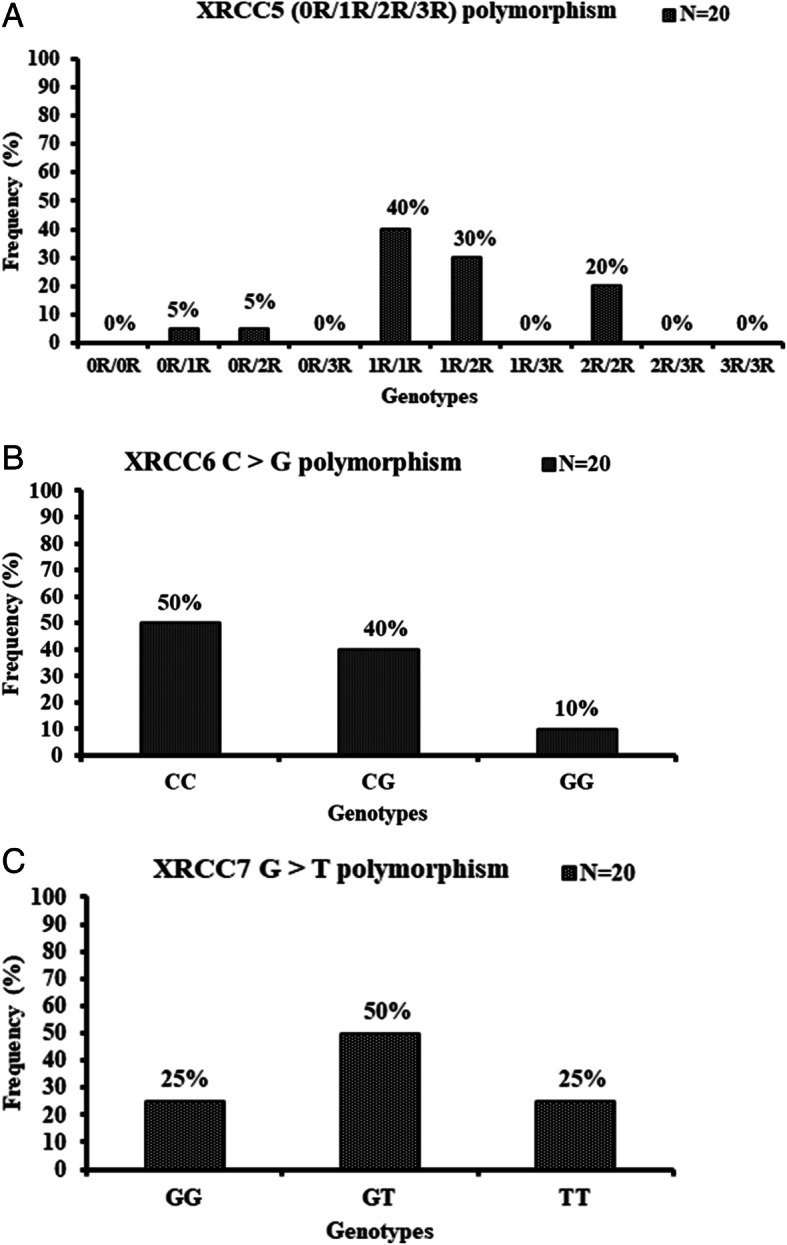
Histogram representing distribution of genotype frequencies of *XRCC5* (0R/1R/2R/3R), *XRCC6* (C/G) and *XRCC7* (G/T) polymorphisms among 20 donors studied (*N* = 20). X-axis represents different genotypes of *XRCC5* (0R/1R/2R/3R) polymorphism and Y-axis represents genotypic frequency (%)

The genotypic frequencies of XRCC5 at 0R/1R, 0R/2R, 0R/3R, 1R/1R, 1R/2R, 1R/3R, 2R/2R and genotypes were 5, 5, 0, 40, 30, 0, 20 and 0%, respectively, among the 20 donors studied (Fig. 2a). Association of polymorphism at *XRCC5* gene with the DNA damage among the 20 donors was studied. For *XRCC5* (0R/1R/2R/3R) polymorphism, five genotypes (0R/1R, 0R/2R, 1R/1R, 1R/2R and 2R/2R) were observed among these 20 donors.

**Fig. 3:**
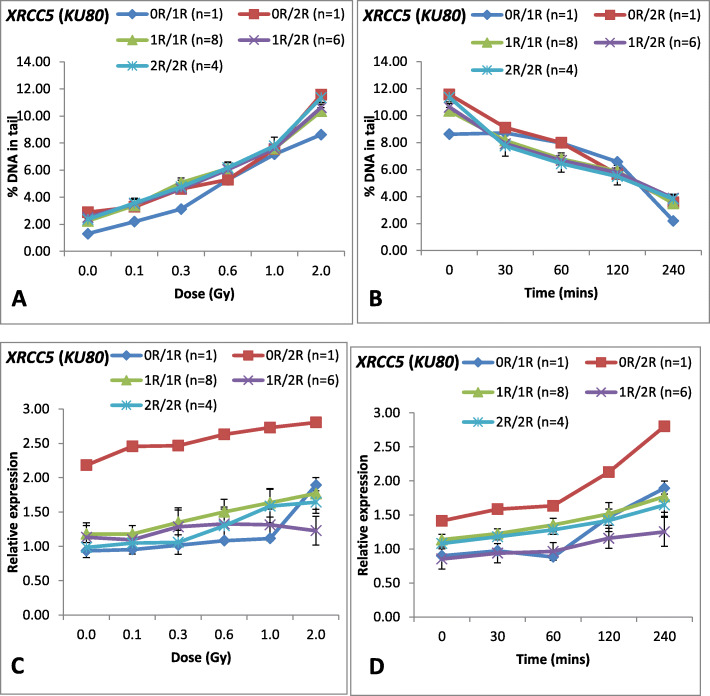
Line graph showing dose response, repair kinetics and mRNA expression of *XRCC5* (*KU80*) among 20 donors and their correlation with each genotype. **a** Dose response curve for DNA damage at different doses for 20 donors. X-axis represents dose (Gy) whereas Y-axis represents DNA damage (Mean ± SEM) in terms of DNA in tail (%). Groups were created amongst 20 donors on the basis of genotypes. **b** Repair kinetics for 20 donors at various post-irradiation time intervals. X-axis represents post-irradiation time (min) whereas Y-axis represents DNA damage (Mean ± SEM) in terms of DNA in tail (%). Groups were created amongst 20 donors on the basis of genotypes. **c** mRNA expression of *XRCC5 (KU80)* at different doses for 20 donors. X-axis represents dose (Gy) and Y-axis represents relative *XRCC5 (KU80)* mRNA expression (Mean ± SEM) at 4 h post-irradiation. Groups were created amongst 20 donors on the basis of genotype. **d** mRNA expression (Mean ± SEM) of *XRCC5 (KU80)* for 20 donors at different post-irradiation time. X-axis represents time (min) and Y-axis represents relative expression. Groups were created amongst 20 donors on the basis of genotype

Dose response of the genotypes at XRCC5 with respect to DNA damage and mRNA expression among the 20 donors is given in Fig. 3a. Regression analysis was performed for each polymorphism with respect to DNA damage and mRNA expression as shown in Table 4. Our results revealed that there is no significant difference in the correlation between DNA damage at various dose points and various genotypes at each gene polymorphism (Table 4). As shown in Table 4, all the genotypes observed at *XRCC5* showed significant positive correlation with DNA damage at different doses studied.

**Table 4:** Correlation coefficient and significance levels for *XRCC5* (0R/1R/2R/3R), *XRCC6* (C/G) and *XRCC7* (G/T) at different biological end points

Gene name	^A^DNA damage at different doses		^B^mRNA expression at 4 h post-irradiation with different doses		^C^DNA repair at different post-irradiation time points		^D^mRNA expression at various post-irradiation time points	
	r	P	r	P	r	P	r	P
***XRCC5*** **(0R/1R/2R/3R)**								
**0R/0R**	0.971^**^	0.001	−0.339	0.512	−0.939^*^	0.018	−0.979^**^	0.004
**0R/2R**	0.986^**^	0.0001	0.846^*^	0.034	−0.900^*^	0.038	0.737^*^	0.019
**1R/1R**	0.947^**^	0.0001	0.464^**^	0.001	−0.859^**^	0.0001	0.366^*^	0.020
**1R/2R**	0.967^**^	0.0001	0.698^**^	0.0001	−0.879^**^	0.0001	0.562^**^	0.001
**2R/2R**	0.916^**^	0.0001	0.831^**^	0.0001	−0.906^**^	0.0001	0.871^**^	0.0001
***XRCC6*** **(C/G)**								
**CC**	0.940^**^	0.0001	0.486^**^	0.0001	−0.892^**^	0.0001	0.364^**^	0.009
**CG**	0.946^**^	0.0001	0.631^**^	0.0001	−0.859^**^	0.0001	0.433^**^	0.005
**GG**	0.970^**^	0.0001	−0.463	0.129	−0.879^**^	0.001	−0.581	0.078
***XRCC7*** **(G/T)**								
**GG**	0.963^**^	0.0001	0.086	0.650	−0.892^**^	0.0001	0.199^*^	0.048
**GT**	0.947^**^	0.0001	−0.020	0.879	−0.868	0.0001	0.100	0.489
**TT**	0.954^**^	0.0001	−0.376^*^	0.041	−0.878^**^	0.0001	−0.290^*^	0.020

Correlation coefficient and significance levels at *XRCC5* (0R/1R/2R/3R), *XRCC6* (C/G) and *XRCC7* (G/T) polymorphism. A. DNA damage at different dose groups, B. mRNA expression at 4 h post-irradiation, C. DNA repair at different post-irradiation time points D. mRNA expression at various post-irradiation time. (^**^) and (^*^) indicates sigificant correlation (*P* < 0.01) and (*P* < 0.05) respectively

Similarly, genotypes of each polymorphism was correlated with DNA repair among these 20 donors (Fig. 3b). As shown in Table 4, no significant difference in the repair pattern was observed among the genotypes observed at all the three gene polymorphisms. All the genotypes showed significant negative correlation with DNA damage and post-irradiation time, indicating no association between *XRCC5* (0R/1R/2R/3R) and repair pattern among these groups (Table 4).

At XRCC5, the mRNA expression level at 0 h and 4 h post irradiation was given in Fig. 3c. As shown in Fig. 3c, variation was observed at dose response of mRNA expression level of *XRCC5* for each genotype. Donors with genotype (0R/0R) showed increasing trend in mRNA expression level at all the doses as compared to other genotypes though not statistically significant (*P* = 0.285). Nevertheless, correlation analysis revealed that genotype (0R/0R) showed decreased *XRCC5* mRNA expression level with increasing doses (*r* = − 0.339, *P* = 0.512), whereas donors with genotype (1R/1R) showed positive correlation (*r* = 0.464, *P* = 0.001) between mRNA expression at *XRCC5* andradiation doses studied. However, donors with genotype 2R allele showed positive correlation between mRNA expression level at *XRCC5* and at different radiation doses studied (0R/2R: *r* = 0.846, *P* = 0.034; 1R/2R: *r* = 0.698, *P* = 0.0001 and 2R/2R: *r* = 0.831, *P* = 0.0001) (Table 4).

At *XRCC5,* mRNA expression was studied at 0 min, 30 min, 60 min, 120 min and 240 min of 2.0 Gy post-irradiation for 20 donors as shown in the line graph (Fig. 3d). As shown in Fig. 3d, genotype (0R/2R) showed increases mRNA expression level with time points as compared to other genotypes though not statistically significant (*P* = 0.406). Correlation analysis revealed variation in the level of mRNA expression of *XRCC5* among the genotypes observed (Table 4). Donor with genotype (0R/0R) showed significant negative correlation (*r* = − 0.979, *P* = 0.004) with mRNA expression and post-irradiation time after 2.0 Gy. Donors with genotype (1R/1R) showed positive correlation (*r* = 0.366, *P* = 0.020) between mRNA expression of *XRCC5* and post-irradiation time points. As observed from the dose response data of *XRCC5* at transcript level, donors having genotype with 2R allele (0R/2R: *r* = 0.737, *P* = 0.019; 1R/2R: *r* = 0.562, *P* = 0.001 and 2R/2R: *r* = 0.871, *P* = 0.0001) showed strong correlation between *XRCC5* mRNA expression and post-irradiation time points as compared to other genotypes.

### *XRCC6* (*KU70*) polymorphism

For the XRCC6 (C/G) polymorphism, the frequencies of the *C/C, C/G*, and *G/G* genotypes were 50, 40, and 10%, respectively, among 20 donors (Fig. 2b). The frequencies of C and G alleles among 20 donors were observed to be 0.7 and 0.3 respectively.

**Fig. 4:**
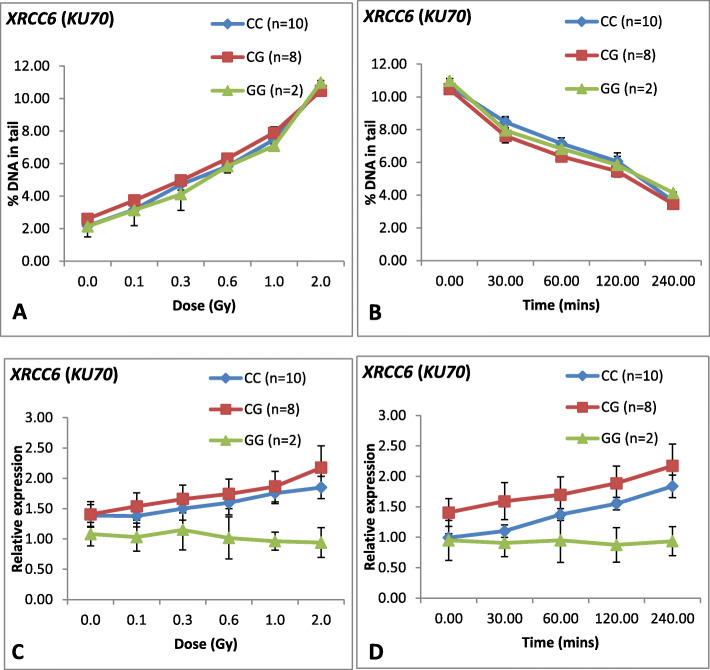
Line graph showing dose response, repair kinetics and mRNA expression of *XRCC6* (*KU70*) among 20 donors and their correlation with each genotype. **a** Dose response curve for DNA damage at different doses for 20 donors. X-axis represents dose (Gy) whereas Y-axis represents DNA damage (Mean ± SEM) in terms of DNA in tail (%). Groups were created amongst 20 donors on the basis of genotypes. **b** Repair kinetics for 20 donors at various post-irradiation time intervals. X-axis represents post-irradiation time (min) whereas Y-axis represents DNA damage (Mean ± SEM) in terms of DNA in tail (%). Groups were created amongst 20 donors on the basis of genotypes. **c** mRNA expression of *XRCC6 (KU70)* at different doses for 20 donors. X-axis represents dose (Gy) and Y-axis represents relative *XRCC6 (KU70)* mRNA expression (Mean ± SEM) at 4 h post-irradiation. Groups were created amongst 20 donors on the basis of genotype. **d** mRNA expression (Mean ± SEM) of *XRCC6 (KU70)* for 20 donors at different post-irradiation time. X-axis represents time (min) and Y-axis represents relative expression. Groups were created amongst 20 donors on the basis of genotype

Association of polymorphism at *XRCC6* gene with respect to DNA damage was assessed among the 20 donors studied. For *XRCC6 (C/G)* polymorphism, *C/C, C/G* and *G/G* polymorphisms were observed among 20 donors. Figure 4a shows, dose response of the above mentioned genotypes among 20 donors studied. Regression analysis showed no significant difference observed among various genotypes in dose response study. As shown in Table 4, all the genotypes of *XRCC6* polymorphism showed significant positive correlation between DNA damage and various doses, thus indicating *XRCC6 (C/G)* polymorphism is not associated with the induction of DNA damage.

Similarly, association of genotypes of *XRCC6* with respect to DNA repair was also studied among these donors. As shown in Fig. 4b, no significant difference was observed in the repair pattern among the genotypes. All the genotypes showed significantly negative correlation between the DNA damage and post-irradiation time points (Table 4).

The mRNA expression level of *XRCC6* was studied at 0 h and 4 h post irradiation. Figure 4c, shows mRNA expression level of *XRCC6* at various doses studied at 4 h post-irradiation. As shown in Fig. 4c, dose response of XRCC6 showed variation in mRNA expression level among the genotypes. Genotypes *C/C* and *C/G* showed positive correlation (*C/C*: *r* = 0.486, *P* = 0.0001 and *C/G*: *r* = 0.631, *P* = 0.0001) between mRNA expression level and radiation doses, whereas *G/G* genotype showed decreasing mRNA expression level with increasing radiation dose (*r* = − 0.463, *P* = 0.129) (Table 4).

Figure 4d showed the line graph of of mRNA expression of *XRCC6* at different time points up to 240 min for 2.0 Gy post-irradiation. Correlation analysis performed at *XRCC6* for mRNA expression in different genotypes is given in Table 4. As evident in Fig. 4d, donors having *C/C* and *C/G* genotypes showed significant positive correlation (C/C: *r* = 0.364, *P* = 0.009 and C/G: *r* = 0.433, *P* = 0.005) between mRNA expression and post-irradiation time points. Donors with *G/G* genotype showed negative correlation (*r* = − 0.581, *P* = 0.078) between mRNA expression and post-irradiation time after 2.0 Gy, which was not statistically significant.

### *XRCC7* (*PRKDC*) polymorphism

**Fig. 5:**
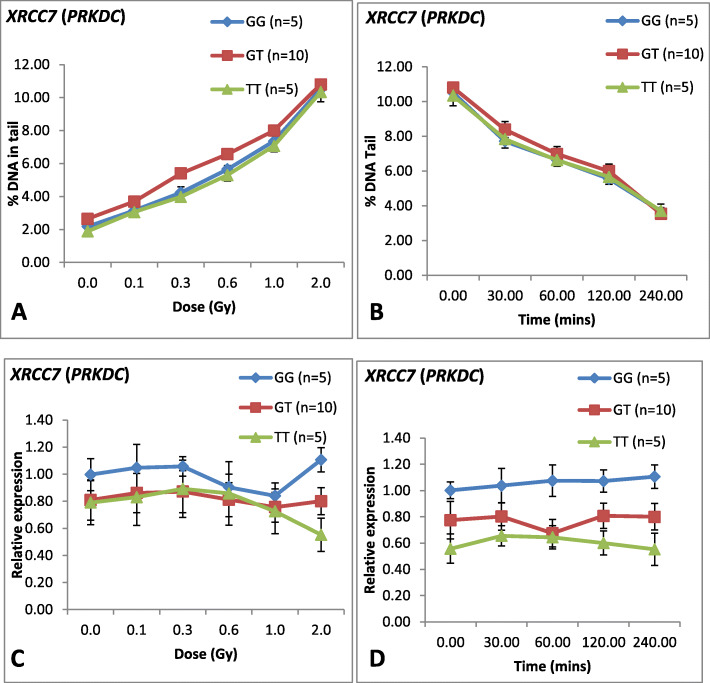
Line graph showing dose response, repair kinetics and mRNA expression of *XRCC7* (*PRKDC*) among 20 donors and their correlation with each genotype. **a** Dose response curve for DNA damage at different doses for 20 donors. X-axis represents dose (Gy) whereas Y-axis represents DNA damage (Mean ± SEM) in terms of DNA in tail (%). Groups were created amongst 20 donors on the basis of genotypes. **b** Repair kinetics for 20 donors at various post-irradiation time intervals. X-axis represents post-irradiation time (min) whereas Y-axis represents DNA damage (Mean ± SEM) in terms of DNA in tail (%). Groups were created amongst 20 donors on the basis of genotypes. **c** mRNA expression of *XRCC7* (*PRKDC*) at different doses for 20 donors. X-axis represents dose (Gy) and Y-axis represents relative *XRCC7* (*PRKDC*) mRNA expression (Mean ± SEM) at 4 h post-irradiation. Groups were created amongst 20 donors on the basis of genotype. **d** mRNA expression (Mean ± SEM) of *XRCC7* (*PRKDC*) for 20 donors at different post-irradiation time. X-axis represents time (min) and Y-axis represents relative expression. Groups were created amongst 20 donors on the basis of genotype

For *XRCC7* G/T polymorphism, the frequencies of the *G/G, G/T* and *T/T* genotypes were 25, 50, and 25%, respectively, among the 20 donors studied (Fig. 2c). The frequencies of G and T alleles among 20 donors were observed to be 0.5 and 0.5 respectively. Association of polymorphism at *XRCC7* gene with the DNA damage among the 20 donors was assessed. Figure 5a showed dose response of the genotypes among 20 donors with respect to DNA damage and mRNA expression level. Regression analysis showed that there was no significant difference among the genotypes observed in the dose response study. As shown in Table 4, all the genotypes of *XRCC7* polymorphism showed significant positive correlation between DNA damage and dose groups studied.

Similarly, association of polymorphism with DNA repair was assessed among these donors. As shown in Fig. 5b, we did not observe any significant difference in the repair pattern among different genotypes. All the genotypes showed significantly negative correlation between the DNA damage and post-irradiation time as shown in Table 4.

The mRNA expression of *XRCC7*was studied at 0 h and 4 h post irradiation. Figure 5c, showed mRNA expression of *XRCC7* at different doses studied at 4 h post-irradiation and inter-individual variation was observed. As shown in Fig. 5c, there was also variation observed in the dose response of *XRCC7* mRNA expression profile with respect to each of the genotypes. The genotypes GG and GT showed no significant correlation (*G/G*: *r* = 0.086, *P* = 0.650 and *G/T*: *r* = − 0.020, *P* = 0.879) between *XRCC7* mRNA expression and radiation doses, whereas genotype *T/T* showed decreasing mRNA expression at *XRCC7* with increasing doses (*r* = − 0.376, *P* = 0.041) (Table 4).

Figure 5d showed the line graph of mRNA expression of *XRCC7* at 0 min, 30 min, 60 min, 120 min and 240 min of 2.0 Gy post-irradiation for 20 donors. Correlation analysis showed variation at the level of mRNA expression of *XRCC7* among the genotypes (Table 4). As observed for dose response of *XRCC7* at transcript level, donors with (*G/G*) and (*G/T*) genotypes showed no significant correlation (*G/G*: *r* = 0.199, *P* = 0.048 and *G/T: r* = 0.100, *P* = 0.489) between mRNA expression of *XRCC7* and post-irradiation time points. Donors with (*T/T*) genotype showed negative correlation (*r* = − 0.290, *P* = 0.020) between mRNA expression of *XRCC7* and post-irradiation time points after 2.0 Gy of irradiation, which was not statistically significant.

### RAR and association of genotypes of adaptive and non-adaptive donors with DNA damage, repair and mRNA expression

**Fig. 6:**
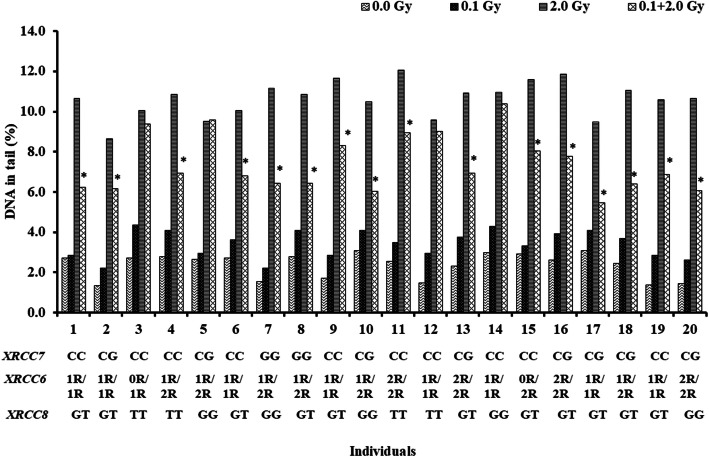
Histogram showing DNA damage of 20 individuals after irradiation of PBMC at priming dose (0.1 Gy) followed by challenging dose (2.0 Gy). (*) indicates significant (*P* < 0.05) decrease in DNA damage. *XRCC5* (3R/2R/1R/0R), *XRCC6*-61C > G and *XRCC7* (G > T) polymorphism for each individual is also shown

RAR study was performed in primed (cells exposed with a priming dose of 0.1 Gy followed by 2.0 Gy after 4 h incubation) and non-primed cells (cells exposed with a 2.0 Gy) among 20 healthy donors. Sixteen of them showed significant reduction of DNA damage in primed cells (cells exposed with a priming dose of 0.1 Gy followed by 2.0 Gy after 4 h incubation) and are called as adaptive donors, whereas four donors did not show any significant reduction of DNA damage and are called as non-adaptive donors. In the present study, emphasis was given to study association of the genotypes of *XRCC5, XRCC6* and *XRCC7* with the adaptive and non-adaptive donors with respect to DNA damage, repair and mRNA expression profile in primed and non-primed cells. Figure 6 is a representation of individual donors showing or not showing radio-adaptive response at DNA damage level.

Table 5 shows average DNA damage at 2.0 Gy, average percentage of repair at 2.0 Gy after 4 h post irradiation and average relative mRNA expression at 2.0 Gy after 4 h post irradiation for *XRCC5, XRCC6* and *XRCC7*. As shown in Table 5, average DNA damage at 2.0 Gy was observed to be significantly higher in non-adaptive donors as compared to adaptive donors for each of the genotypes of the polymorphisms studied. However, there was no significant difference in average DNA damage, repair and mRNA expression level among the genotypes of XRCC5, XRCC6 and *XRCC7* polymorphisms among each group (adaptive or non-adaptive donors). But the repair percentage was comparatively higher among non-adaptive donors.

**Table 5:** Comparison of genotypes with various end points studied. Average DNA damage (%T) for 2.0 Gy at 0 h and 4 h post irradiation (after 4 h) and relative expression (for 2.0 Gy after 4 h) in PBMCs of adaptive and non-adaptive donors

Genotypes	Adaptive individuals (***N*** = 16)				Non-adaptive individuals (***N*** = 4)			
	(N, frequency %)	Average %T at 2.0 Gy (0 h) ± SD	Average %T at 2.0 Gy (after 4 h) ± SD	Average relative expression at 2.0Gy (after 4 h) ± SD	(N, frequency %)	Average %T at 2.0Gy (0 h) ± SD	Average %T at 2.0 Gy (after 4 h) ± SD	Average relative expression at 2.0 Gy (after 4 h) ± SD
***XRCC5 (0R/1R/2R/3R)***								
**0R/1R**	0 (0.00%)	0.00 ± 0.00	0.00 ± 0.00	0.00 ± 0.00	1 (25.00%)	9.36 ± 0.00	2.20 ± 0.00	0.79 ± 0.00
**0R/2R**	0 (0.00%)	0.00 ± 0.00	0.00 ± 0.00	0.00 ± 0.00	1 (25.00%)	10.37 ± 0.00	3.58 ± 0.00	1.12 ± 0.00
**1R/1R**	7 (43.75%)	6.81 ± 1.03	3.43 ± 0.53	1.11 ± 0.30	1 (25.00%)	8.98 ± 0.00	3.91 ± 0.00	1.12 ± 0.00
**1R/2R**	5 (31.25%)	6.42 ± 0.32	4.11 ± 0.10	1.04 ± 0.13	1 (25.00%)	9.55 ± 0.00	2.92 ± 0.00	1.04 ± 0.00
**2R/2R**	4 (25.00%)	7.41 ± 1.23	3.81 ± 0.73	1.27 ± 0.28	0 (0.00%)	0.00 ± 0.00	0.00 ± 0.00	0.00 ± 0.00
***XRCC6 (C/G)***								
**CC**	7 (43.75%)	7.42 ± 0.98	3.80 ± 0.59	1.24 ± 0.40	3 (75.0%)	9.57 ± 0.72	3.23 ± 0.91	1.03 ± 0.24
**CG**	7 (43.75%)	6.38 ± 0.75	3.54 ± 0.55	1.27 ± 0.22	1 (25.0%)	9.55 ± 0.00	2.92 ± 0.00	0.95 ± 0.00
**GG**	2 (12.50%)	6.42 ± 0.01	4.14 ± 0.08	1.10 ± 0.04	0 (0.0%)	0.00 ± 0.00	0.00 ± 0.00	0.00 ± 0.00
***XRCC7 (G/T)***								
**TT**	4 (25.00%)	6.62 ± 0.95	3.87 ± 0.32	0.83 ± 0.24	1 (25.0%)	9.55 ± 0.00	2.92 ± 0.00	0.82 ± 0.00
**TG**	10 (62.50%)	6.72 ± 0.82	3.55 ± 0.57	1.08 ± 0.24	1 (25.0%)	10.37 ± 0.00	3.58 ± 0.00	1.14 ± 0.00
**GG**	2 (12.50%)	7.92 ± 1.42	4.34 ± 0.36	1.05 ± 0.06	2 (50.0%)	9.17 ± 0.27	3.05 ± 1.21	1.02 ± 0.04

“N” indicates number of donors showing a particular genotype, *SD* Standard Deviation

But when compared between adaptive and non-adaptive donors in non-primed cells at 2.0 Gy, the genotypes 1R/2R of *XRCC5* showed similar mRNA expression among the adaptive vs non-adaptive donors (1.04 ± 0.13 vs 1.04 ± 0.0). There was also significant difference (*p* < 0.05) in the percentage of DNA damage (%T) among adaptive vs non-adaptive donors. At *XRCC6* polymorphism, donors with *C/C* and *C/G* genotypes showed significantly higher (*P* < 0.05) mRNA expression level among adaptive donors as compared to non-adaptive donors (*C/C*: 1.24 ± 0.40 vs1.03 ± 0.24; *C/G*: 1.27 ± 0.22 vs 0.95 ± 0.00). At *XRCC7*, all the three genotypes (*TT, TG* and *GG*) did not show significant mRNA expression level among adaptive vs non-adaptive donors as shown in Table 5.

Distribution of genotypes at *XRCC5, XRCC6* and *XRCC7*, average DNA damage and relative expression in primed cells (adaptive donors) and non-primed cells (non-adaptive donors) are given in Table 6. As shown in Table 6, average DNA damage in primed cells (cells exposed with 0.1 Gy followed by a challenge dose of at 2.0 Gy) showed significantly higher percentage of DNA damage (%T) among non-adaptive donors as compared to adaptive donors for each of the genotypes of the polymorphisms studied. At *XRCC5*, the genotypes 1R/1R and 1R/2R showed no significant difference in mRNA expression level in primed cells of adaptive vs non adaptive donors (1R/1R: 1.09 ± 0.31 vs 1.12 ± 0.00; 1.04 ± 0.13 vs 1.04 ± 0.0). However, at *XRCC6*, the donors with C/G genotype showed significantly increased (*P* < 0.05) mRNA expression in primed cells among adaptive vs non-adaptive donors (C/G: 1.22 ± 0.26 vs 0.95 ± 0.00). It showed significantly higher % of DNA damage in primed cells of non-adaptive donors as compared to adaptive donors. At *XRCC7*, mRNA expression is marginally higher in primed cells of adaptive donors as compared to non-adaptive donors for G/G genotype. But higher % of DNA damage was observed among non-adaptive donors as compared to adaptive donors.

**Table 6:** Genotype frequency distribution, DNA damage (%T), and average relative expression in PBMCs among adaptive and non-adaptive donors exposed to priming dose (0.1 Gy) followed by a challenge dose (2.0 Gy)

Genotypes	Adaptive individuals (***N*** = 16)			Non-adaptive individuals (***N*** = 4)		
	(N, Frequency %)	Average %T ± SD in primed cells	Average relative expression ± SD in primed cells	(N, Frequency %)	Average %T ± SD in primed cell	Average relative expression ± SD in primed cells
***XRCC5*** **(0R/1R/2R/3R)**						
**0R/1R**	0 (0.00%)	0.00 ± 0.00	0.00 ± 0.00	1(25.00%)	9.36 ± 0.00	0.92 ± 0.00
**0R/2R**	0 (0.00%)	0.00 ± 0.00	0.00 ± 0.00	1(25.00%)	8.01 ± 0.00	1.14 ± 0.00
**1R/1R**	7 (43.75%)	7.15 ± 1.67	1.09 ± 0.31	1(25.00%)	8.98 ± 0.00	1.12 ± 0.00
**1R/2R**	5 (31.25%)	6.44 ± 0.32	1.04 ± 0.13	1(25.00%)	9.55 ± 0.00	1.04 ± 0.00
**2R/2R**	4 (25.00%)	7.41 ± 1.23	1.27 ± 0.28	0 (0.00%)	0.00 ± 0.00	0.00 ± 0.00
***XRCC6*** **(C/G)**						
**CC**	7 (43.75%)	7.27 ± 0.96	1.24 ± 0.40	3 (75.0%)	7.71 ± 1.45	1.14 ± 0.22
**CG**	7 (43.75%)	6.90 ± 1.69	1.22 ± 0.26	1 (25.0%)	9.55 ± 0.00	0.95 ± 0.00
**GG**	2 (12.50%)	6.42 ± 0.01	1.10 ± 0.04	0 (0.0%)	0.00 ± 0.00	0.00 ± 0.00
***XRCC7*** **(G/T)**						
**GG**	4 (25.00%)	6.35 ± 0.43	1.16 ± 0.11	1 (25.0%)	9.55 ± 0.00	0.95 ± 0.00
**GT**	10 (62.50%)	7.08 ± 1.41	1.27 ± 0.37	1 (25.0%)	8.01 ± 0.00	1.35 ± 0.00
**TT**	2 (12.50%)	7.92 ± 1.42	1.05 ± 0.14	2 (50.0%)	7.56 ± 2.02	1.04 ± 0.17

“N” indicates number of donors showing a particular genotype, *SD* Standard Deviation

## Discussion

DSBs are one of the most lethal DNA lesions induced by IR, as well as endogenous reactive oxygen species (ROS) [34], which are mainly repaired through non-homologous end joining (NHEJ) repair pathway in circulating lymphocytes. Ku proteins (Ku70 and Ku80) proteins play crucial role in NHEJ repair. Ku is a heterodimer composed of 69kD and 83 kD polypeptides, which initiates DSB repair process by binding to the broken DNA ends and recruits the DNA-PK catalytic subunit (DNA-PKcs) to form the active DNA-PK enzyme. The active DNA-PK enzyme, through its kinase activity recruit other enzymes, such as Artemis, that process and join the broken ends [35]. Ku proteins also take part in DNA replication, apoptotic signaling and telomere maintenance [36]. Ku70 and Ku80 genes are very active during G_0_/G_1_ and early S-phase of the cell cycle [37–39].

DDR induced by genotoxic stress including IR lead to alteration of gene expression profile in human cells [17, 18]. Several studies have demonstrated early and late responses of miRNA expression of several genes involved in various cellular and molecular processes in human cells in response to IR. Transcriptional profiling of DNA repair genes is used as a biomarker for radiation exposure and has been used to gain insight into the molecular mechanisms induced by low dose exposures in a variety of cell types for e.g. cell cultures of human myeloid cells [40–42]. However, limited studies are available, where transcriptional expression of NHEJ repair genes have been studied in peripheral blood mononuclear cells. Earlier, we had demonstrated dose response, RAR and time dependent studies which have shown alteration in transcript profile of NHEJ genes in resting PBMCs [21]. Post irradiation changes at mRNA transcription level at early time points provides information on transcriptional regulation and DNA repair response in human PBMC exposed to IR. In the present study, post irradiation time point changes were quantitated at mRNA level up to 240 min (4 h), which showed individual variation. Similarly, DNA damage and repair kinetics among 20 donors have also shown high degree of inter-individual variation. Hence, it is interesting to correlate NHEJ polymorphism of XRCC5, XRCC6 and XRCC7 with DNA damage and mRNA expression.

Several studies have been carried out to find out the role of cellular activities involving the action of DNA repair and cell cycle checkpoints [43–45]. Individual sensitivity to IR is a key cellular phenomenon for DNA repair and signaling of cell cycle checkpoints. Several studies have been carried out on DDR, DNA repair and cell cycle checkpoints [43, 46, 47]. Individual variation may contribute towards radio-sensitivity in a population [48]. Radio-resistance helps cells to repair DNA damage efficiently [49]. In the present study, some of the genotypes of NHEJ polymorphisms showed association with increased mRNA expression at XRCC5, XRCC6 and XRCC7.

Some of the DNA repair gene polymorphism such as XPC, XRCC1 has shown significant correlations between genotypes and induced DNA damages. For instance, at *XPC (*Lys939Gln and Ala499Val) polymorphism where the haplotypes “T-A” (in the order of Ala499Val-PAT-Lys939Gln) was associated with the lowest DNA damages, thus suggesting that the DNA repair capacity of host cells might be modulated by specific *XPC* polymorphisms [30]. Similarly, Cornetta et al.*,* reported that polymorphisms in *XRCC1* DNA repair genes could influence individual DNA repair capacity [50]. However, limited data is available regarding the association of DNA polymorphism with DNA damage, repair and mRNA expression at NHEJ genes among healthy donors.

The promoter of *XRCC5* contains seven copies of *cis* elements, which are essential for basal expression and are involved in CpG methylation [51]. Studies pertaining to this predicted that this VNTR polymorphism, which includes a variable number of Sp1-binding motifs, that might be influencing the transcriptional activity of *XRCC5,* which lead to a phenotypic variation that could affect susceptibility to cancer [22]. Furthermore, fewer tandem repeats in the promoter of *XRCC5* was associated with enhanced levels of the *XRCC5* protein in bladder cancer patients [22]. However, DNA damage and repair kinetics among these groups were not significantly different. In the present study, association between *XRCC5* (3R/2R/1R/0R) polymorphism and its mRNA expression showed significant positive correlation for (2R) allele at various doses and post-irradiation time intervals.

There are reports which show that *XRCC6*-61C/G polymorphism is associated with an increased risk of cancers, including breast cancer and gliomas [27]. Our results on *XRCC6* (C/G) polymorphism studies indicate significant positive correlation of the *XRCC6* transcript level with various irradiation doses as well as post-irradiation time for wild type allele (C), however, *XRCC6* transcript level was negatively correlated for variant (G) allele. Accordingly, radiation induced DNA damage was observed to be lower in donors with polymorphic group containing (C) allele as compared to the polymorphic group containing (G) allele. Consequently, DNA repair was faster in donors with polymorphic group containing (C) allele as compared to the polymorphic group containing (G) allele. All these findings indicate that wild type (G) allele may be associated with the radio-sensitivity and DNA repair capacity of an individual.

DNA polymorphism at G6721T of *XRCC7* (rs7003908) is located in the intron 8 of the gene and may play a role in regulating splicing and therefore cause mRNA instability [52, 53]. NHEJ is the major pathway for DSB repair in human cells [54]. Few studies have shown association of G6721T polymorphism of *XRCC7* with several types of cancers [28, 55–60]. Our results on *XRCC7* (G/T) polymorphism indicate no significant correlation of the *XRCC7* transcript level with different dose groups as well as post-irradiation time for wild type allele (G). However, *XRCC7* transcript level was negatively correlated for variant (T) allele. Despite these variations in the transcript levels, the radiation induced DNA damage and its repair was not significantly different among these genotypes.

In summary, our studies on association of *XRCC5* (3R/2R/1R/0R), *XRCC6* (C/G) and *XRCC7* (G/T) polymorphism with DNA damage, repair capacity and its mRNA expression showed that wild type (G) allele may be associated with the radio-sensitivity and DNA repair capacity of an individual. At the transcript level, (2R) allele of *XRCC5* polymorphism and (T) allele of *XRCC7* polymorphism also showed significant association at various doses of IR and post-irradiation time intervals.

It is quite intriguing that individual variation due to intrinsic DNA repair capacity decides the fate of a person’s disease status. While DNA repair assays are required to be considered as potential clinical tools for prevention or treatment of disease of an individual [10], genetic variation studies provide important information towards radio-sensitivity as well as susceptibility towards a disease through DNA repair gene polymorphism. Hence, it is necessary to conduct genetic variation studies involving multiple DNA repair pathways to find out susceptibility of individual capacity to overcome the radio-sensitivity during radio-therapeutic treatments. It will be therefore important to integrate high throughput assays such as genome wide association studies, SNP profiling using next generation sequencing, global transcriptome, methylome and proteomic profiles along with restriction enzyme based SNP/VNTR assays to identify suitable biomarkers for radiation exposure in health care system to reach to the clinics as well as population based studies.

The present study demonstrated that radio-adaptive experiments showed changes at transcript level among adaptive donors in many of the genotypes as compared to non-adaptive donors. However, further studies at post-translational modification and proteomics level are required to understand the cause of genetic variability. Radio-sensitivity is an important aspect for patients undergoing radio-therapeutic treatments as efficacy of chemotherapy as well as radiation therapy depends upon individual response, which can decide treatment dosages and time span of treatment required. The present study also assessed the role of NHEJ repair gene polymorphism in RAR.

## Conclusions

DNA DSBs are highly deleterious and involvement of NHEJ repair gene polymorphism are of high importance for genome integrity. Further, high throughput studies on larger number of gene polymorphisms may be helpful for studying radio-sensitivity studies. NHEJ repair gene polymorphisms play an important role in RAR and thus can be used as potential biomarkers to identify radiosensitive and radio-resistant individuals in a population as well as among cancer patients undergoing radio-therapeutic treatments.

## Data Availability

Data will be available on special request to corresponding author.
